# Coccidioidomycosis Incidence in Arizona Predicted by Seasonal Precipitation

**DOI:** 10.1371/journal.pone.0021009

**Published:** 2011-06-20

**Authors:** James D. Tamerius, Andrew C. Comrie

**Affiliations:** School of Geography and Development, University of Arizona, Tucson, Arizona, United States of America; University of Cincinnati, United States of America

## Abstract

The environmental mechanisms that determine the inter-annual and seasonal variability in incidence of coccidioidomycosis are unclear. In this study, we use Arizona coccidioidomycosis case data for 1995–2006 to generate a timeseries of monthly estimates of exposure rates in Maricopa County, AZ and Pima County, AZ. We reveal a seasonal autocorrelation structure for exposure rates in both Maricopa County and Pima County which indicates that exposure rates are strongly related from the fall to the spring. An abrupt end to this autocorrelation relationship occurs near the the onset of the summer precipitation season and increasing exposure rates related to the subsequent season. The identification of the autocorrelation structure enabled us to construct a “primary” exposure season that spans August-March and a “secondary” season that spans April–June which are then used in subsequent analyses. We show that October–December precipitation is positively associated with rates of exposure for the primary exposure season in both Maricopa County (R = 0.72, p = 0.012) and Pima County (R = 0.69, p = 0.019). In addition, exposure rates during the primary exposure seasons are negatively associated with concurrent precipitation in Maricopa (R = −0.79, p = 0.004) and Pima (R = −0.64, p = 0.019), possibly due to reduced spore dispersion. These associations enabled the generation of models to estimate exposure rates for the primary exposure season. The models explain 69% (p = 0.009) and 54% (p = 0.045) of the variance in the study period for Maricopa and Pima counties, respectively. We did not find any significant predictors for exposure rates during the secondary season. This study builds on previous studies examining the causes of temporal fluctuations in coccidioidomycosis, and corroborates the “grow and blow” hypothesis.

## Introduction

Coccidioidomycosis (also known as “Valley Fever”) is a non-communicable febrile respiratory disease caused by the inhalation of arthroconidia (i.e., spores) from the fungi *Coccidioides spp.* [1], [2]. The fungi reside in warm, arid and semi-arid soils of the Americas [3]. Approximately 40% of *Coccidioides spp.* infections are symptomatic and can result in severe complications such as community acquired pneumonia (CAP), lung cavities, and disseminated infections that can affect the central nervous system, skin, bones, joints and other organs [4]. Risk factors for severe infections include race, age, and immunosuppression [2], [5], [6]. Approximately 29% (95% confidence interval: 16%, 44%) of diagnosed CAP cases in Tucson, Arizona are related to *Coccidioides spp.* infection [7].

It has long been speculated that environmentally mediated mechanisms cause significant seasonal and inter-annual fluctuations in the incidence of coccidioidomycosis infection [8]. One proposed mechanism suggests that precipitation modifies the suitability of the environment for fungal growth. Consistent with this hypothesis are several relationships identified between case rates and precedent precipitation in Arizona [8]–[10]. In addition to influencing the presence of *Coccidioides spp.* in the soil, the absence of precipitation may cause the fungus to sporulate and aerosolize more readily. Several studies point to a negative association between the seasonality of cases and precipitation as support of this claim [8], [9]. Together, these mechanisms constitute the “grow and blow” hypothesis: precipitation facilitates the growth of the fungus, while subsequent dry conditions result in sporulation and enable the spores to become airborne, ultimately resulting in human exposures [11]. Yet, despite extensive study, there is currently no clear-cut and ecologically consistent link identified between the environment and coccidioidomycosis rates. This is likely due in part to noisy case data of inadequate temporal extent, and the absence of a simple method for identifying the fungus in the soil and describing its spatial and temporal variability.

Here we use 12 years of laboratory-confirmed Arizona case data for Maricopa and Pima counties to investigate patterns of coccidioidomycosis and relate these to climate conditions. This is the first study to examine relationships between coccidioidomycosis and climate both in Maricopa and Pima counties. This study improves on previous studies conducted in Arizona by using a more straightforward and accurate adjustment to account for the duration between exposure and disease reports. We also examine autocorrelation patterns in exposure rates to improve our understanding of the seasonality of the disease. We investigate the relationships between case rates and several climate variables using lag correlation and regression techniques to corroborate the “grow and blow” hypothesis. Finally, we create a multivariate model that can be used to predict variability of coccidioidomycosis rates in Pima and Maricopa 9–16 months in advance.

## Materials and Methods

Case data were obtained from the Arizona Department of Health Services (ADHS) for January 1995 through December 2006. Case data that did not indicate the diagnosis county (<1%) or diagnosis date (∼10%) were removed. A total of 18,954 cases in Maricopa County and 4,645 cases in Pima County were included in the study. These counties encompass the two major Arizona cities of Phoenix and Tucson where the vast majority of coccidioidomycosis cases occur.

We determined monthly case incidence per 100,000 of the general population for both Maricopa and Pima counties using linearly interpolated annual population estimates provided by the US Census Bureau. The date of exposure for each diagnosed case was estimated using the results of an enhanced surveillance study conducted in 2007 by ADHS. The enhanced surveillance indicated that the median time from symptom-onset-to-diagnosis was 55 days and an average of 209 days [12]. To account for the symptom-onset-to-diagnosis lag we subtracted 54 days (median lag provided to us from preliminary analysis of the ADHS study, prior to final publication [12]) from the diagnosis date of each case to estimate the time of symptom onset. We then subtracted 14 days from the estimated symptom onset date to account for the incubation period which generally lasts 1–4 weeks [13]. Case data were aggregated by month since it is unlikely that the date of exposure can be accurately estimated at a finer resolution; this also improves the signal-to-noise ratio. Counties were modeled separately since climate effects may differ between regions. This resulted in a monthly time series of estimated *Coccidioides spp.* exposure rates for Pima and Maricopa counties.

**Figure 1:**
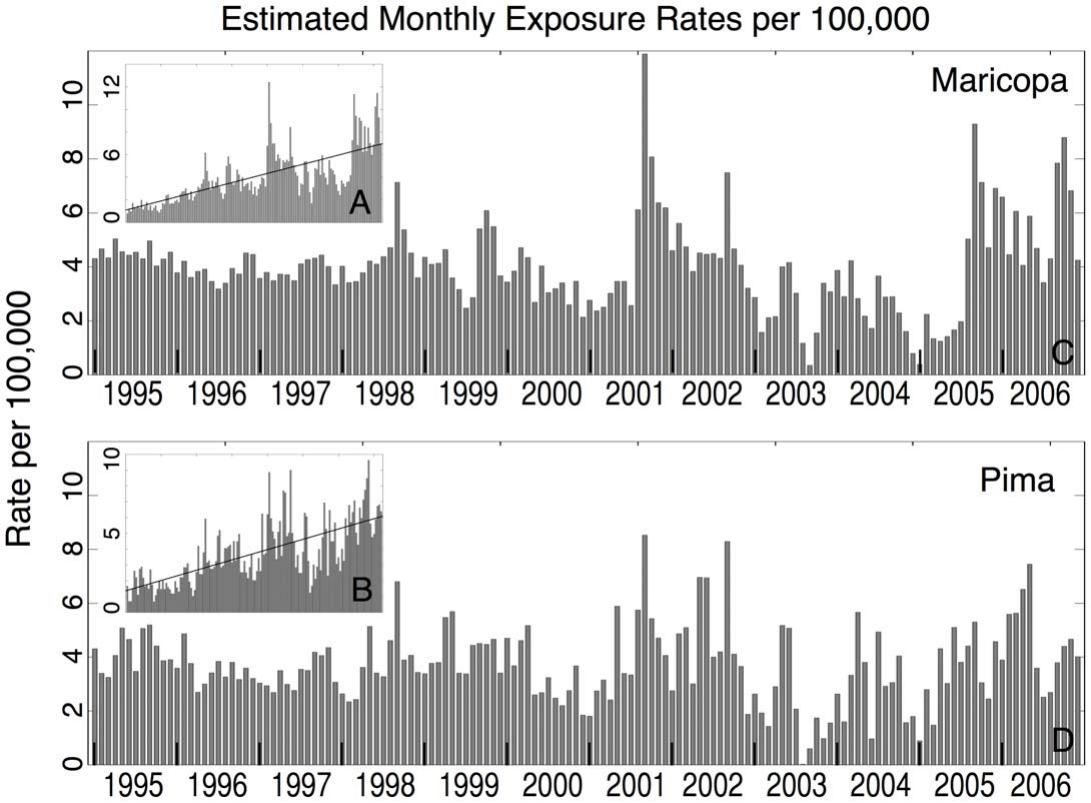
Estimated exposure rates. Estimated exposure rates per 100,000 population from January 1995 through December 2006. The plots describe the crude estimated exposure rates with best-fit line for (a) Maricopa County and (b) Pima County. We also show the detrended estimates of exposure rates with the average crude exposure rate added for (c) Maricopa County and (d) Pima County.

The strong increasing linear trend in exposure rates observed across the study period (see Figure 1A and 1B) cannot easily be attributed to climatic fluctuations [11]. Since the observed trend will bias potential relationships between the climate conditions and exposure rates, the trend was removed by subtracting a best-fit line from the exposure time series (i.e. “detrended”) (see figure 1C and 1D). The average exposure rate from the time series of mean raw exposure rates (i.e. not detrended) was added to the detrended exposure rates to equalize the average between the detrended and raw time series.

Daily weather data were retrieved from the Arizona Meteorological Network (AZMET) for all available stations in the population centers of Maricopa County (Phoenix; n = 3) and Pima County (Tucson; n = 1). Variables included temperature, relative humidity, wind speed, mean wind vector, soil temperature (2 and 4 inch depths), vapor pressure deficit, precipitation, solar radiation, and heat units. The soil temperature variables were discarded due to large inconsistencies in the signal over time. The remaining data were aggregated by county and monthly climatic averages were generated. This resulted in a monthly time series for each climate variable and county.

Monthly precipitation data from all available National Weather Service stations in Phoenix (n = 10) and Tucson (n = 5) were used in preference to AZMET precipitation data since a greater number of stations were available. Air quality data that describes the concentration of particles of 10 micrometers or less (PM10) were obtained from Maricopa County Air Quality Department (n = 10) and Pima County Department of Environmental Quality (n = 7). PM10 data has been hypothesized to be a strong proxy for windblown spore concentrations, and seasonal variability in case rates of coccidioidomycosis have been shown to be correlated with seasonal variations in PM10 [9]. The data were aggregated by county and monthly averages were calculated, as for the climate data.

We investigated the seasonality of exposure rates in Maricopa and Pima counties by examining the exposure rates for each month over the study period. To further examine seasonality, we evaluated the autocorrelation structure of the exposure rates by correlating each month with all other months up to 12 months prior:
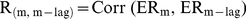
(1)where ER_m_ is a vector of exposure rates per 100,000 population for month m (e.g., January), ER_m-lag_ is a vector of exposure rates per 100,000 population for month m minus a lag that varied between 1 (e.g., December) and 12 (e.g., previous January) months, and R is Pearson's correlation coefficient. This resulted in a 12×12 “lag correlation matrix” which was used to generate an image plot so that the correlation between each combination of months could be assessed. This lag correlation matrix showed a strong seasonal autocorrelation structure in exposures which enabled us to define a “primary” exposure season spanning August-March and a “secondary” exposure season spanning April–June (see Results for justification of these groupings) . We averaged exposure rates across these seasons, generating time series of average seasonal exposure rates. Because the primary exposure season spans the New Year, only 11 primary exposure seasons (i.e. 1995/1996–2005/2006) were generated from the 12 years of data.

To capture both concurrent and long-lead influences on the disease ecology, we investigated associations between monthly exposure rates and concurrent and preceding environmental variability by creating a bivariate lag correlation matrix:
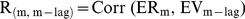
(2)where EV_m-lag_ is a time series of an environmental variable (e.g., precipitation) for month m minus a lag that varied between 0 and 36 months. This resulted in 36×12 lag correlation matrices for each of the 12 environmental variables derived for our study, which were then used to generate an image plot for each county. Visual examination of the image plots and correlation statistics indicated precipitation yielded the strongest relationships with exposure rates; other climate variables showed weaker or inconsistent relationships to exposure rates. Thus, subsequent analysis focused on relationships between precipitation and exposure rates and we only report on these relationships hereafter.

The correlation between average monthly exposure rates for the primary and secondary season and precipitation up to 36 months prior were then calculated:
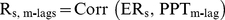
(3)where PPT_m-lag_ is a time series of precipitation for month m (the last month in the season) minus a lag that varied between 0 and 36 months. This showed statistically significant associations in both counties between exposure rates during the primary season and concurrent and antecedent monthly precipitation approximately 1 year prior. No significant relationships were observed between precipitation and the secondary season.

Identification of the most promising statistical relationships between precipitation and exposure rates during the primary season enabled the production of a multivariate regression model to estimate the number of exposures during the fall exposure season. The model inputs include October–November (previous year; “grow”) and September-March (concurrent; “blow”) precipitation to estimate exposure rates for August-March:
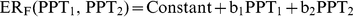
(4)where ER_F_ is a vector of average fall exposure rates for August-March from 1995–2006 (n = 11), PPT_1_ is a vector indicating the total precipitation (mm) for October–December for 1994–2005 (n = 11), and PPT_2_ is the total precipitation (mm) for August-March from 1995–2006 (concurrent with exposure period; n = 11).

## Results

The estimated exposure rate increased substantially between 1995 and 2006 in both Maricopa and Pima counties. Over all study years, the average monthly exposure rate for both Maricopa and Pima were approximately 4 per 100,000 population (Figure 1A and 1B). During 1995 Maricopa County averaged approximately 1 exposure per 100,000 population per month, increasing to approximately 7 exposures per 100,000 per population per month by the end of 2006. Similarly, in Pima County the average exposure rate increased from 1 to 6 exposures per 100,000 population from January 1995 to December of 2006. The data were detrended to account for the increasing trend in exposures observed during the study period. The average exposure rate from the time series of mean raw exposure rates (i.e. not detrended) was added to the detrended exposure rates to equalize the average between time series. Thus, the detrended exposure rates should be treated with caution. Further, the variance of the exposure rates increased strongly with time in both counties, especially after 2000 (Figure 1). This variance was not removed by detrending and has implications for subsequent statistical analyses (Figures 1C and 1D; see Discussion).

In general, monthly exposure rates between counties are highly correlated throughout the study period (R = 0.64; p = 0.0001). Periods of elevated exposure (defined here as exposure rates greater than 6 per 100,000 population) occurred in both counties during the primary exposure season of 1998, 2001, and 2002. In addition, Maricopa County had independent periods of elevated exposure during the primary season in 2005 and 2006, whereas Pima County had independent periods of elevated exposure during the secondary season in 2001, 2002 and 2006.

**Figure 2:**
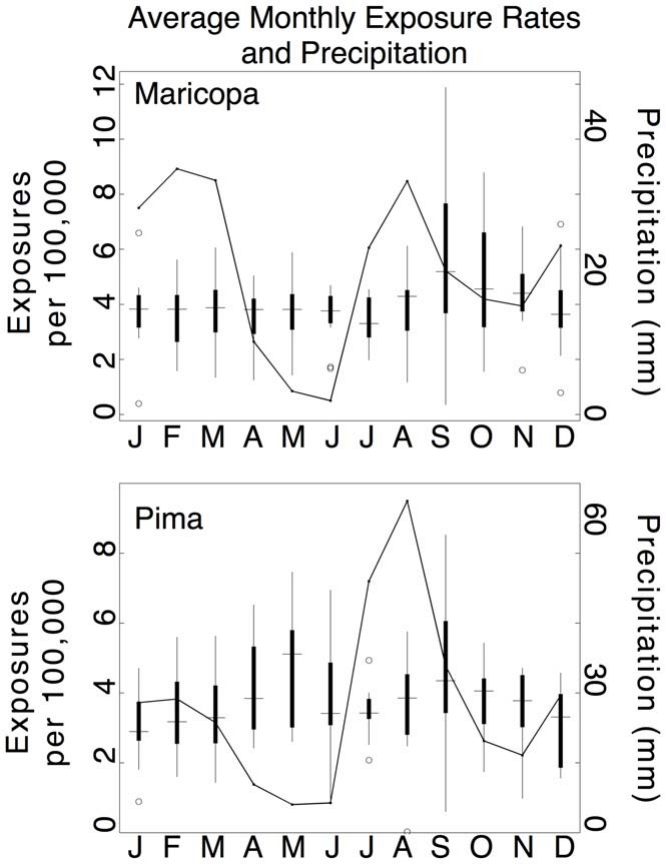
The seasonality of exposure rates. Box plots of monthly exposure rates for Maricopa (top) and Pima (bottom) counties. The central hash mark is the median, the edges of the box indicate the 25th and 75th percentiles, the whiskers extend to the most extreme data points not considered outliers, and the “o” markers indicate outliers. The plots show the bimodal nature of exposure rates in Pima County, with peaks occurring in May and September. In Maricopa County, only a fall peak in September is observed. The average seasonal precipitation is also showed (black line).

A seasonal signal characterizes the exposures rates in both counties (Figure 2). On average, exposures for Maricopa peak at approximately 6 per 100,000 population in September, decline rapidly to approximately 4 exposures per 100,000 by December, and then gradually decline through July prior to a slight increase in exposures in August. In Pima County, exposure rates are characterized by two peaks, one in May and the other in September. The average exposure rate for May and September are nearly identical with approximately 5 exposures per 100,000 population for each month. Exposure rates are minimal between the peaks, in January (∼3 exposures per 100,000 population) and July (∼3.5 exposures per 100,000 population). For both counties, the highest exposure rates observed during the study period occurred in September of 2001, with 12.5 and 9.5 exposures per 100,000 population in Maricopa and Pima counties, respectively.

**Figure 3:**
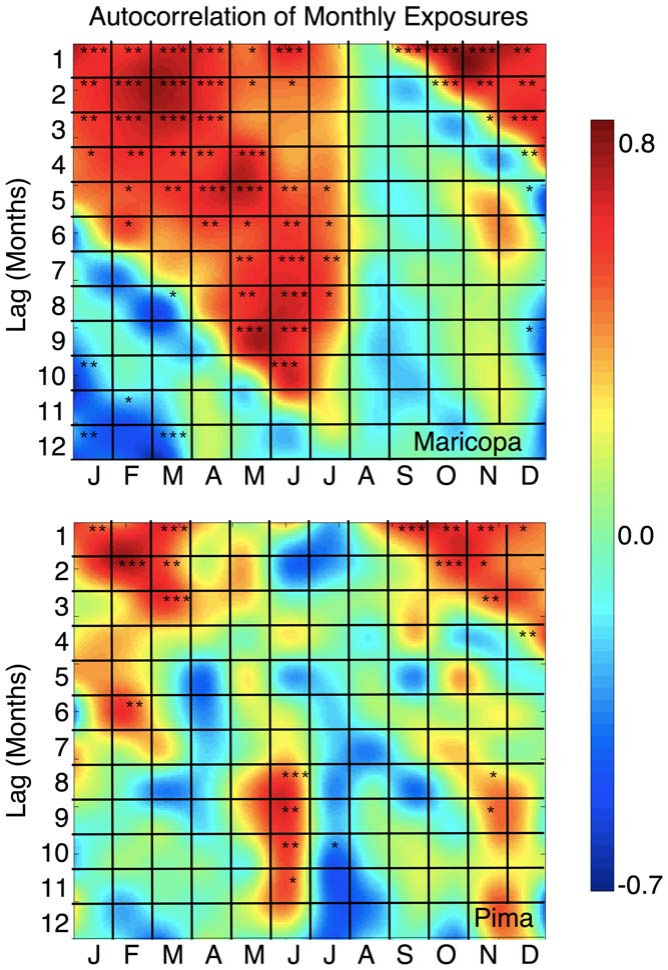
Autocorrelation of exposure rates. These plots indicate the autocorrelation of exposure rates in Maricopa (top) and Pima (bottom) counties. The x-axis is the month of the year, and the y-axis is the lag in months. The surface color indicates the Pearson's correlation coefficient (R) for each combination of months going back one year. The stars indicate the significance level of individual R at the 0.10 (*), the 0.05 (**) and the 0.01 (***). A large wedge of positive correlations in Maricopa beginning in August and lasting through July are shown, suggesting that exposure rates during these months are regulated by the same mechanism. Pima County shows a similar pattern from August-March, but the correlations from April–June become inconsistent.

Exposure rates are significantly autocorrelated in time at approximately 3–4 month lags in both counties (not shown). However, autocorrelation is not equal across all months of the year (Figure 3). In Maricopa County, exposure rates are positively correlated from September-July, but this association ends abruptly in August. In Pima County, there are similar relationships but they are not as strong and have less structure than those in Maricopa County. Specifically, autocorrelations of exposure rates in Pima County are positive between August-March becoming inconsistent from April–June. In July, Pima County exposure rates are not correlated with any of the twelve previous months, comparable to August in Maricopa County. The autocorrelation structures of both counties suggested that the exposure rates from August-March are likely dependent on the same mechanism. Thus, the exposure rates for August-March months were aggregated to simplify analysis and increase the strength of the signal. This period from August-March is referred to as the “primary” exposure season since it is the only season of elevated exposures that is consistent across counties. We also define a “secondary” exposure season that spans April–June which is associated with the elevated exposure rates during these months in Pima County. Although an increase in exposure rates during April–June is nearly non-existent in Maricopa, we generated a time series of exposure rates for the secondary season here for consistency with Pima County. Since July does not naturally fall into the primary or secondary exposure season and a minimal number of exposures are observed during this month, we do not include it in either season.

We examined all antecedent and concurrent precipitation-exposure relationships for Maricopa and Pima. We determined there are two significant associations between the fall exposure season and precipitation. First, accumulated precipitation during October–December is significantly correlated with exposure rates during the subsequent primary exposure season (i.e. August-March) in both Maricopa (R = 0.72, p = 0.012) and Pima (R = 0.69, p = 0.019). A leave-one-out jackknife method was used to demonstrate that the association is not strongly dependent upon a single observation. In Maricopa, the jackknife analysis resulted in an R_max_ = 0.80 (p = 0.005), and R_min_ = 0.61 (p = 0.061), whereas in Pima the R_max_ = 0.76 (p = 0.010) and the R_min_ = 0.57 (p = 0.086). In addition to the relationship with precedent precipitation, exposure rates during the primary exposure season are negatively correlated with concurrent precipitation in both Maricopa (R = −0.79, p = 0.004) and Pima (R = −0.64, p = 0.019) counties. The leave-one-out jackknife analysis for Maricopa indicated an R_min_ = −0.87 (p = 0.001) and R_max_ = −0.71 (p = 0.023). In Pima, the jackknife analysis indicated an R_min_ = −0.75 (p = 0.013) and R_max_ = −0.51 (p = 0.134). We were unable to identify a significant relationship between precipitation and the secondary exposure season in either Maricopa or Pima counties.

**Figure 4:**
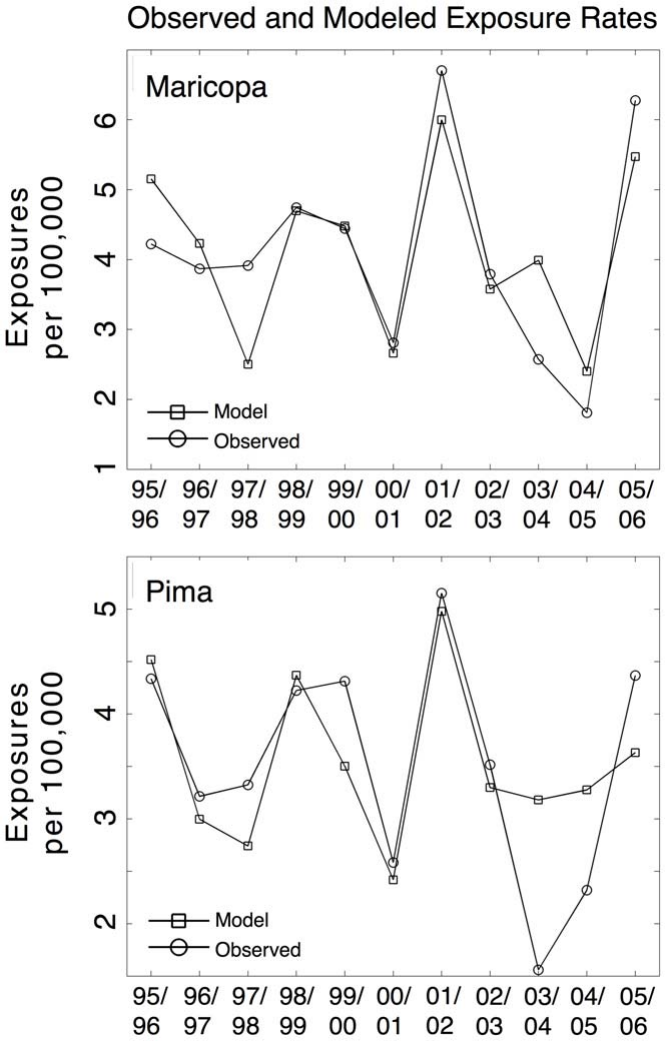
Observed versus modeled exposure rates. Observed and modeled exposure rates for Maricopa (top) and Pima (bottom) counties per 100,000 population. The exposure rates are the average monthly exposure rates for August-March. The model inputs include October–December precipitation (mm) from the previous year (“grow”) and concurrent August-March precipitation (“blow”).

The relationships between precipitation and exposure rates during the primary coccidioidomycosis exposure season enabled the production of a linear regression model (Equation 3; Table 1). The Maricopa model explains 69% (p = 0.009) of the variance in the study period, with an adjusted R^2^ = 0.62. The Pima model explains 54% (p = 0.045) of the variance with an adjusted R^2^ = 0.42 (Table 1; Figure 4).

**Table 1:** Results of the linear regression model (Equation 3) for Maricopa County and Pima County.

Predictor	Coefficient(95% CI)	P-Value	R^2^	Adjusted R^2^	P-Value
Maricopa County					
Constant	5.24(2.33, 8.15)	0.003	0.69	0.62	0.01
Oct–Dec Precipitation	0.012(−0.01, 0.03)	0.23			
Aug-Mar Precipitation	−0.01(−0.02, 0.001)	0.06			
Pima County					
Constant	3.86(0.99, 6.73)	0.02	0.54	0.42	0.05
Oct–Dec Precipitation	0.01(−0.01, 0.03)	0.17			
Aug-Mar Precipitation	−0.005(−0.02, 0.01)	0.33			

The models indicate that inter-annual variation in exposure rates during the period of August-March are significantly associated by concurrent and antecedent precipitation during the study period.

**Figure 5:**
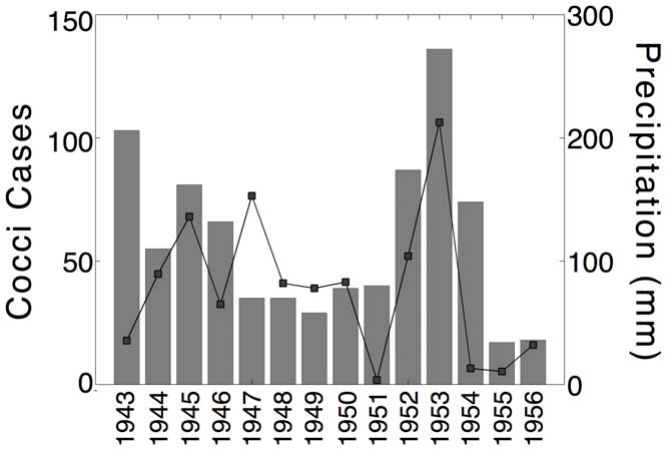
Validation of model. This figure shows the case rates (left y-axis) in Hugenholtz (1957) and the corresponding October–December precipitation (right y-axis) the previous year. Unfortunately, the coccidioidomycosis data were aggregated across the calendar year, making it difficult to assess the impacts of antecedent and concurrent precipitation on case rates.

A weakness of this study is that only 12 years of data were available, limiting confidence in the statistical associations identified. To address this weakness we examined independent data on coccidioidomycosis cases at Williams Air Force Base in Maricopa County for 1943–1956 [8]. Using PRISM precipitation data [14] for Williams Air Force Base we were able to show that October–December precipitation is significantly correlated with case rates for the subsequent calendar year (R = 0.50, p = 0.068; Figure 5). However, since these data were aggregated across the calendar year instead of the exposure seasons as defined herein, it is difficult to assess the importance of this result. Further, this relationship is strongly dependent upon 1953 since case rates and the corresponding 1952 October–December precipitation were 234% and 271% of average (both maxima), respectively. Finally, the seasonality of these coccidioidomycosis data are strongly bimodal [8], in contrast to the single peak that is present in the ADHS data reported herein, making comparisons even more difficult.

## Discussion

A data set of laboratory-confirmed coccidioidomycosis cases for 12 years has enabled the production of a time series of predicted *Coccidioides spp.* exposure. An enhanced surveillance study by ADHS was used to estimate exposure dates and resulted in a slightly modified characterization of the seasonality of *Coccidioides spp.* exposure in Pima County relative to that previously reported [9]. Further, a consistent linear increase in exposures over the study period, which has been reported previously [15] was removed from the time series. This trend may be due to increased surveillance and reporting [16], or soil disturbance caused by construction [15].

Similarities and contrasts in the seasonality of exposure rates were observed between Maricopa and Pima counties. Both counties observed an increase in exposure rates beginning in August with maximum exposure rates observed in September followed by a gradual decline into January. In Maricopa average monthly exposure rates remained relatively consistent for January–July. In Pima, on the other hand, exposures began to rise in February and a second peak was observed in May with minimal rates observed in July between the May and September exposure maxima (Figure 2). The bimodal seasonality in Pima County is consistent with a previous study [9]. Although Maricopa County did occasionally experience significant increases in exposure rates during the secondary exposure season (e.g., 2001 and 2003), these were less substantial and less consistent relative to those observed in Pima County. The absence of a consistent secondary peak in Maricopa County is unexpected since it was observed in an earlier study [8] and because the secondary peak is pronounced in Pima County which has very similar climate (Figure 2).

Lag correlation analyses indicated a strong seasonal autocorrelation structure in exposures for both counties. This is especially true for Maricopa County where exposures from August-June are highly correlated with each other. In other words, if the rate of exposure is greater than average during September, the monthly rate of exposures will likely be above average for each month through June of the following year. In Pima County, autocorrelation is strong for August-February. These autocorrelation structures suggest that the fungal spores released during the late-summer/fall “bloom” (i.e. period when the fungus sporulates) may persist in the environment and continue to infect humans for several months. In turn, this implies the intensity of the fall bloom is the primary factor determining the number of exposures through the winter and into the spring. An abrupt end in the monthly autocorrelation of exposures occurs in July/August (Figure 3) which coincides with the summer precipitation season. This suggests that the end of the season may be due to the suppressing effects of precipitation on the aerosolization of spores. Another possibility is that during July/August the effects of a new crop of spores on exposures is beginning to exceed the effects of the previous year's crop, thus obscuring any relationship from the previous season. Although Pima County has a similar autocorrelation structure to that in Maricopa County, autocorrelation in Pima County diminishes in April–June before disappearing completely in July. This discrepancy between autocorrelation structures may be due to the increases in exposure rates in Pima during the secondary exposure season, thereby interfering with the association. This is supported by the fact that the months in the middle of the secondary peak (i.e., April and May) are not significantly correlated with any of the previous 12 months. Yet in June, when rates have declined notably from May, exposure rates are again related to those from the previous fall (Figures 2 and 3).

Precipitation in October–December is positively correlated with exposure incidence during the following August-March (i.e., 8–16 months later). This relationship is consistent across both Maricopa and Pima, explaining 61% and 45% of the variance for detrended monthly exposure rates from 1995–2006, respectively. It is unclear what environmentally-mediated mechanisms potentially link October–December precipitation and human exposure to the fungal spores 8–16 months later. It is possible that the precipitation does not act directly on the fungus, but catalyzes events in the environment that produce favorable conditions months afterward. Though rodent-borne coccidioidomycosis has not previously been considered for the maintenance of *C. spp.*, this long delay in response to precipitation hints at the possibility for a rodent reservoir host. Similar to the trophic cascade hypothesis associated with variable outbreaks of plague and hantavirus, high precipitation during the preceding winter may result in an increase in rodent populations [17]. This mechanism potentially increase the density of rodent carcasses the following fall, which have been hypothesized to be suitable environments for fungal growth due to their high nutrient content [18]. Furthermore, the relationship between exposure rates and concurrent precipitation during the months of August-March is negative. This is consistent with the hypothesis that precipitation suppresses spore aerosolization and, conversely, that dry soils enhance spore aerosolization. However, October–December precipitation was negatively correlated with September-March precipitation of the following year (∼one year later) during the study period in both Maricopa (R = −0.69) and Pima (R = −0.65). This correlation made it difficult to assess the effects of the “grow” and “blow” precipitation mechanisms independently. Thus, it is possible that only one of these mechanisms is affecting the epidemiology of the disease. Attempts to separate the independent effects of these mechanisms by examining individual months and outliers were inconclusive.

Studies in Kern County, California, have not shown a significant relationship between coccidioidomycosis rates and precipitation [19]. Differences between findings in California and Arizona may be a result of different precipitation patterns in the Central Valley versus Maricopa and Pima counties; difference in the physiology of the fungi since two difference species, *Coccidioides immitis* and *Coccidioides posadasii*, are the causal agents of coccidioidomycosis in the Central Valley and Arizona, respectively; and soil characteristics between locations may also modify the effects of precipitation on soil moisture [19].

One weakness of this study is that the variance of the estimated exposure rates increase with time in both counties. Our assumption is that this is not due to climatic changes. This creates statistical problems since seasons that occur later in the study period will have more leverage thereby influencing statistical associations more than those of earlier seasons. In an attempt to ameliorate the effects of this increasing trend in variance, we equalized the variance across the time series and found that the statistical associations between October–December precipitation and August-March become marginally stronger. However, for simplicity, we only reported the results from the basic detrended time series.

In all, this study builds on previous studies examining the causes of fluctuations in coccidioidomycosis case rates in Arizona, and directly corroborates the “blow and grow” hypothesis [11]. Although this study investigated natural factors involved in the dispersion of spores across large spatial scales, it is possible that human factors such as construction may also play a role [4]. Further specification of environmentally-mediated relationships will likely require highly-resolved data of longer duration or a reliable and rapid method for systematic sampling of *Coccidioides* spores in the soil or air.

## References

[1] Fisher MC, Koenig GL, White TJ, Taylor JT (2002). Molecular and phenotypic description of Coccidioides Posadasii sp nov., previously recognized as the non-California population of Coccidioides immitis.. Mycologia.

[2] Pappagianis D (1988). Epidemiology of coccidioidomycosis.. Current Topics in Medical Mycology, Vol 2.

[3] Fisher FS, Bultman MW, Johnson SM, Pappagianis D, Zaborsky E (2007). Coccidioides Niches and habitat parameters in the southwestern United States.. Ann NY Acad Sci.

[4] Park BJ, Sigel K, Vaz V, Komatsu K, McRill C (2005). An epidemic of coccidioidomycosis in Arizona associated with climatic changes, 1998–2001.. J Infect Dis.

[5] Ampel NM (2007). Coccidioidomycosis in persons infected with HIV-1.. Ann NY Acad Sci.

[6] Mosley D, Komatsu K, Vaz V, Vertz D, England B (1996). Coccidioidomycosis - Arizona, 1990–1995.. MMWR.

[7] Valdivia L, Nix D, Wright M, Lindberg E, Fagan T (2006). Coccidioidomycosis as a common cause of community-acquired pneumonia.. Emerg Infect Dis.

[8] Hugenholtz P (1957). Climate and coccidioidomycosis.. Proceedings of the Symposium on Coccidioidomycosis, Phoenix, Arizona. Publication 575.

[9] Comrie AC (2005). Climate Factors Influencing Coccidioidomycosis Seasonality and Outbreaks.. Env Hlth Persp.

[10] Kolivras KM, Comrie AC (2003). Modeling valley fever (coccidioidomycosis) incidence on the basis of climate conditions.. Int J Biometeorol.

[11] Comrie AC, Glueck MF (2007). Assessment of climate-coccidioidomycosis model: model sensitivity for assessing climatologic effects on the risk of acquiring coccidioidomycosis.. Ann NY Acad Sci.

[12] Tsang CA, Anderson SM, Imholte SB, Erhart LM, Chen S (2010). Enhanced surveillance of coccidioidomycosis, Arizona, USA, 2007–2008.. Emerg Infect Dis.

[13] Chin J (2000). Control of Communicable Disease Manual..

[14] WestMap: Western Climate Mapping Initiative (2010). Climate Analysis and Modeling.. http://www.cefa.dri.edu/Westmap/.

[15] Sunenshine RH, Anderson S, Erhart L, Vossbrink A, Kelly PC (2007). Public health surveillance for coccidioidomycosis in Arizona.. Ann NY Acad Sci.

[16] Tabor JA, O'Rourke MK (2010). A risk factor study of coccidioidomycosis by controlling differential misclassifications of exposure and susceptibility using a landscape ecology approach.. Sci Tot Env.

[17] Brown J, Morgan-Ernest SK (2002). Rain and rodents: complex dynamics of desert consumers.. BioScience.

[18] Sharpton TJ, Stajich JE, Rounsley SD, Wortman JR (2009). Comparative genomic analyses of the human fungal pathogens Coccidioides and their relatives.. Genome Res.

[19] Talamantes J, Behseta S, Zender C (2007). Fluctuations in Climate and Incidence of Coccidioidomycosis in Kern County, California: A Review.. Ann NY Acad Sci.

